# CREBH-FGF21 axis improves hepatic steatosis by suppressing adipose tissue lipolysis

**DOI:** 10.1038/srep27938

**Published:** 2016-06-15

**Authors:** Jong-Gil Park, Xu Xu, Sungyun Cho, Kyu Yeon Hur, Myung-Shik Lee, Sander Kersten, Ann-Hwee Lee

**Affiliations:** 1 Department of Pathology and Laboratory Medicine, Weill Cornell Medical College, New York, USA; 2 BCMB Allied Program, Weill Cornell Medical College, New York, USA; 3 Department of Medicine, Samsung Medical Center, Sungkyunkwan University School of Medicine, Seoul, Korea; 4 Severance Biomedical Science Institute and Department of Internal Medicine; Yonsei University College of Medicine; Seoul, Korea; 5 Nutrition, Metabolism and Genomics Group, Division of Human Nutrition, Wageningen University, The Netherlands

## Abstract

Adipose tissue lipolysis produces glycerol and nonesterified fatty acids (NEFA) that serve as energy sources during nutrient scarcity. Adipose tissue lipolysis is tightly regulated and excessive lipolysis causes hepatic steatosis, as NEFA released from adipose tissue constitutes a major source of TG in the liver of patients with nonalcoholic fatty liver diseases. Here we show that the liver-enriched transcription factor CREBH is activated by TG accumulation and induces FGF21, which suppresses adipose tissue lipolysis, ameliorating hepatic steatosis. CREBH-deficient mice developed severe hepatic steatosis due to increased adipose tissue lipolysis, when fasted or fed a high-fat low-carbohydrate ketogenic diet. FGF21 production was impaired in CREBH-deficient mice, and adenoviral overexpression of FGF21 suppressed adipose tissue lipolysis and improved hepatic steatosis in these mice. Thus, our results uncover a negative feedback loop in which CREBH regulates NEFA flux from adipose tissue to the liver via FGF21.

Nonalcoholic fatty liver disease (NAFLD) comprises a spectrum of liver disorders from simple steatosis, which is characterized by excess accumulation of triglyceride (TG) within hepatocytes, to nonalcoholic steatohepatitis (NASH) that accompanies hepatocyte death, liver inflammation and fibrosis^1^,^2^. NAFLD is closely associated with obesity and insulin resistance, and is considered as the hepatic manifestation of the metabolic syndrome. NAFLD is the most common liver disease in industrialized countries, and recognized as a global public health problem due to its rising prevalence in parallel with the epidemic obesity^3^.

Hepatic steatosis is caused by an imbalance in TG homeostasis in the liver. Increased flow of fatty acids to hepatocytes from adipose tissues or from the diet, increased de novo lipogenesis, decreased oxidative disposal of fatty acids, or impaired export of TG as very low density lipoprotein (VLDL) is expected to result in a net increase in hepatic TG content^4^. The relative contributions by these factors to human NAFLD have yet to be clearly defined. Interestingly, a study has demonstrated that nonesterified fatty acids (NEFA) that originate from adipose tissues account for the majority of hepatic TG in NAFLD patients^5^, implicating that dysregulation of adipose tissue lipolysis may critically contribute to fatty liver diseases. Consistent with this notion, it has been shown that disruption of lipolytic enzymes or the regulatory proteins of lipolysis strongly impacts TG deposition in the liver^6^,^7^,^8^,^9^. Adipose tissue lipolysis is tightly regulated by neuroendocrine signals, among which catecholamines and insulin are known to play central roles. β-adrenergic stimulation activates adipose triglyceride lipase (ATGL) and hormone-sensitive lipase (HSL) via the cAMP/protein kinase A (PKA) signaling pathway, leading to enhanced lipolysis in white adipocytes^10^. PKA directly phosphorylates HSL and perilipin-1, a lipid droplet (LD)-associated protein, increasing HSL activity and its translocation onto LD surface^11^,^12^,^13^,^14^. PKA-phosphorylation of perilipin-1 also stimulates ATGL, as CGI-58, an important activator of ATGL is released from the phosphorylated perilipin-1^15^,^16^. In contrast, insulin potently inhibits both basal and catecholamine-stimulated lipolysis by activating phosphodiesterase-3B that decreases cAMP level, and consequently represses HSL in the fed state^10^.

Cyclic AMP-responsive element-binding protein H (CREBH, encoded by Creb3l3) is a bZiP transcription factor that is highly expressed in the liver^17^,^18^. CREBH is synthesized as an ER precursor form and proteolytically activated by Golgi-localized proteases. These site 1 and site 2 proteases liberate the N-terminal portion of CREBH, allowing it to act as a transcriptional activator^19^. CREBH is emerging as a critical regulator of glucose and lipid metabolism^20^. It has been shown that CREBH is transcriptionally activated by PPARα and the glucocorticoid receptor^21^,^22^, and promotes gluconeogenesis^22^, plasma TG clearance^17^, and lipid droplet formation in the liver^23^. It has been also reported that CREBH deficient mice are susceptible to hepatic steatosis when fasted^17^, or fed an atherogenic Paigen diet^24^. However, the underlying mechanism and the precise role of CREBH in hepatic TG homeostasis remained unknown or controversial. Zhang and colleagues demonstrated that a group of genes involved in de novo lipogenesis, fatty acid elongation, fatty acid oxidation were down-regulated in the liver of CREBH deficient mice fed the Paigen diet compared to WT controls^24^. However, it was unexplored whether this gene expression change impacts TG accumulation in CREBH deficient liver, or reflects the consequence of severe steatosis in these mice.

In this study, we determined the source of TG accumulated in the liver of CREBH deficient mice, and explored the mechanism by which CREBH controls TG homeostasis in the liver. We demonstrate that CREBH-deficient mice develop severe hepatic steatosis, when fasted or fed a high-fat low-carbohydrate ketogenic diet (KD), conditions in which fatty acids serve as major energy source. We find that adipose tissue lipolysis is marked increased in CREBH-deficient mice, suggesting that the increased flow of NEFA from adipose tissue to the liver is primarily responsible for the liver steatosis in CREBH deficient mice. In contrast, de novo lipogenesis, fatty acid oxidation, and VLDL secretion are largely unaffected in CREBH deficient liver. We also demonstrate that fibroblast growth factor (FGF) 21 is a critical CREBH target that ameliorates hepatic steatosis. FGF21 is a member of FGF family mainly secreted by the liver^25^,^26^ and acts as a metabolic regulator exerting various metabolic benefits, including weight loss and improvement of glucose homeostasis^27^,^28^,^29^,^30^,^31^,^32^. We find that hepatic FGF21 expression correlates well with the degree of steatosis in various rodent models, and CREBH plays a critical role in FGF21 expression. Severe hepatic steatosis and inflammation in KD-fed CREBH deficient mice were substantially reversed by adenoviral overexpression of FGF21. Coupled with our previous finding that CREBH is activated in fatty livers^23^, we propose that CREBH plays a central role in hepatic TG homeostasis by inducing FGF21, which suppresses NEFA flux to the liver, thereby ameliorating hepatic steatosis.

## Results

### CREBH deficiency increases hepatic TG due to increased adipose tissue lipolysis

**Figure 1:**
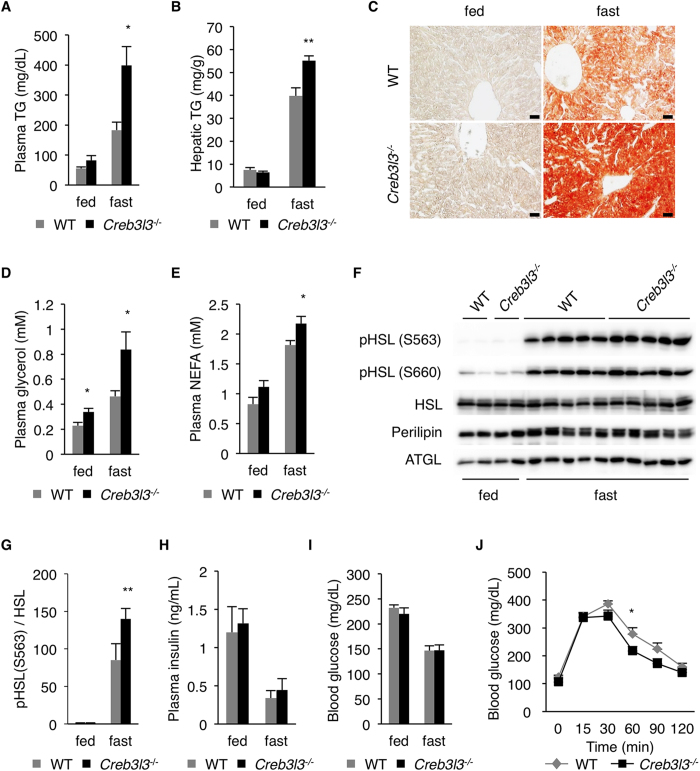
CREBH deficiency exacerbates fasting-induced hepatic steatosis by increasing adipose tissue lipolysis. (**A**) Plasma TG levels measured at fed state or after a 16 h fast (n = 5 per group). (**B**) Hepatic TG levels (n = 6–9 per group). (**C**) Oil red O staining of liver sections. Scale bar, 200 μm. (**D**) Plasma glycerol, and (**E**) NEFA levels (n = 6 per group). (**F**) HSL and perilipin phosphorylation, and ATGL expression in WAT measured by western blotting. (**G**) Quantification of Ser-563 phospho-HSL levels normalized to total HSL. (**H**) Plasma insulin and (**I**) blood glucose levels (n = 6 per group). (**J**) Intraperitoneal glucose tolerance test (GTT) (n = 6 per group). Data are shown as mean ± s.e.m. *P < 0.05, **P < 0.01.

CREBH deficient (Creb3l3^−/−^) mice fed normal chow diet exhibited increased plasma and hepatic TG levels compared with WT mice, when measured after a 16 h fast (Fig. 1A,B). Oil red O staining of frozen liver sections also showed higher amount of neutral lipids in Creb3l3^−/−^ hepatocytes than in WT controls (Fig. 1C). In contrast, plasma and hepatic TG levels in the fed state were not significantly different between WT and Creb3l3^−/−^ mice (Fig. 1A–C).

To determine the cause of severe fasting-induced hepatic steatosis in Creb3l3^−/−^ mice, we first asked whether adipose tissue lipolysis was increased in these mice. Prolonged fasting increased plasma glycerol and NEFA levels in both WT and Creb3l3^−/−^ mice, indicating enhanced adipose tissue lipolysis during nutrient scarcity. Interestingly, Creb3l3^−/−^ mice exhibited significantly higher fasting plasma glycerol and NEFA levels than the WT mice (Fig. 1D,E), suggesting that the loss of CREBH further increased adipose tissue lipolysis. Consistent with this, HSL phosphorylation at protein kinase A (PKA) sites (Ser-563 and Ser-660) in WAT was significantly increased in Creb3l3^−/−^ compared to the WT mice in the fasted state^33^ (Fig. 1F,G, Supplementary Fig. 1A). Perilipin phosphorylation as revealed by the band shift in western blot^34^ was also increased in Creb3l3^−/−^ compared to WT mice (Fig. 1F, Supplementary Fig. 1A). ATGL abundance tended to be higher in Creb3l3^−/−^ WAT than in WT controls (Fig. 1F, Supplementary Fig. 1A), but this difference did not reach statistical significance. Insulin is a critical regulator of adipose tissue lipolysis, suppressing lipase activity through the Akt kinase-PKA pathway^35^. Fasting decreased plasma insulin and glucose levels in both WT and Creb3l3^−/−^ mice to similar degrees (Fig. 1H,I). Creb3l3^−/−^ mice exhibited a modestly improved glucose tolerance (Fig. 1J), excluding the possibility that the increased lipolysis in Creb3l3^−/−^ mice might be due to low insulin level or compromised insulin action. These data suggest that CREBH regulates adipose tissue lipolysis in an insulin-independent manner.

**Figure 2:**
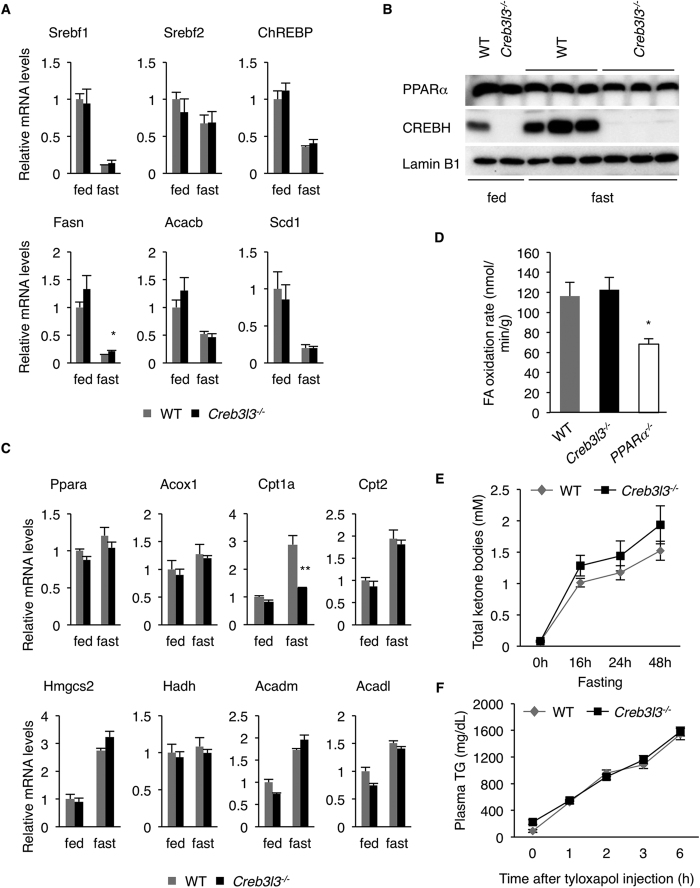
De novo lipogenesis, VLDL secretion, lipid oxidation, and ketogenesis in Creb3l3^−/−^ mice. (**A**) Hepatic mRNA levels for each gene were determined by qRT-PCR (n = 4 per group). (**B**) Liver nuclear extracts were subjected to western blotting using antibodies against PPARα, CREBH and Lamin B1. (**C**) Hepatic mRNA levels of lipid oxidation-related genes determined by qRT-PCR (n = 4 per group). (**D**) Fatty acid oxidation activity in liver lysates (n = 3 per group). (**E**) Total plasma ketone bodies during fasting (n = 6 per group). (**F**) VLDL secretion assay (n = 6 per group). Data are shown as mean ± s.e.m. *P < 0.05.

We next investigated whether de novo lipogenesis, VLDL secretion rate, fatty acids oxidation, and ketogenesis were altered in Creb3l3^−/−^ mice, contributing to the increased hepatic steatosis. We found comparable expression of major lipogenic transcription factors, Srebf1, Srebf2, and ChREBP, and their target genes, such as Fasn, Acacb, and Scd1 between WT and Creb3l3^−/−^ mice in the fed state (Fig. 2A). Srebf1 and its target genes were markedly downregulated by fasting in both WT and Creb3l3^−/−^ mice, likely reflecting normal insulin-mediated regulation of these genes. In contrast, fasting-induced expression of Apoa4, a representative CREBH target gene was markedly downregulated in Creb3l3^−/−^ liver (Supplementary Fig. 1B), consistent with previous report^23^. Furthermore, overexpression of the constitutively active CREBH(N) had no effect on TG synthesis in primary mouse hepatocytes using glycerol and oleic acid as substrates (Supplementary Fig. 1C), suggesting that CREBH has minimal role in lipogenesis. The scavenger receptor CD36 involved in fatty acid uptake was similarly expressed between WT and Creb3l3^−/−^ livers (Supplementary Fig. 1B). The abundance of PPARα, the central transcriptional regulator of fatty acid oxidation and ketogenesis, was comparable between WT and Creb3l3^−/−^ livers (Fig. 2B). Consistently, mRNA levels of PPARα target genes, such as Acox1, Cpt2, Hmgcs2, Hadh, Acadm, and Acadl were similar between WT and Creb3l3^−/−^ livers, except Cpt1a which appeared to be regulated by both CREBH and PPARα (Fig. 2C). Western blotting also confirmed normal expression of Hmgcs2 protein, the rate-limiting enzyme for ketogenesis, in Creb3l3^−/−^ liver (Supplementary Fig. 1D). Importantly, fatty acid oxidation capacity was comparable between WT and Creb3l3^−/−^ liver lysates (Fig. 2D). Creb3l3^−/−^ mice exhibited slightly higher fasting plasma ketone levels compared with the WT mice, likely reflecting the increased flow of fatty acids to the liver to serve as ketogenic substrates (Fig. 2E). Consistent with this scenario, *in vitro* ketone production rate was comparable between WT and Creb3l3^−/−^ primary hepatocytes, when octanoic acids were provided as substrate (Supplementary Fig. 1E). Similarly, overexpression of CREBH(N) had no effect on ketone production in primary hepatocytes (Supplementary Fig. 1F). Finally, there was no noticeable difference in the hepatic VLDL-TG production rate between WT and Creb3l3^−/−^ mice (Fig. 2F). Taken together, these data suggest that the severe fasting-induced hepatic steatosis in Creb3l3^−/−^ mice is primarily due to increased adipose tissue lipolysis, whereas de novo lipogenesis, VLDL production, and fatty acid oxidation were unaffected in Creb3l3^−/−^ mice.

### Severe hepatic steatosis of CREBH deficient mice on ketogenic diet

**Figure 3:**
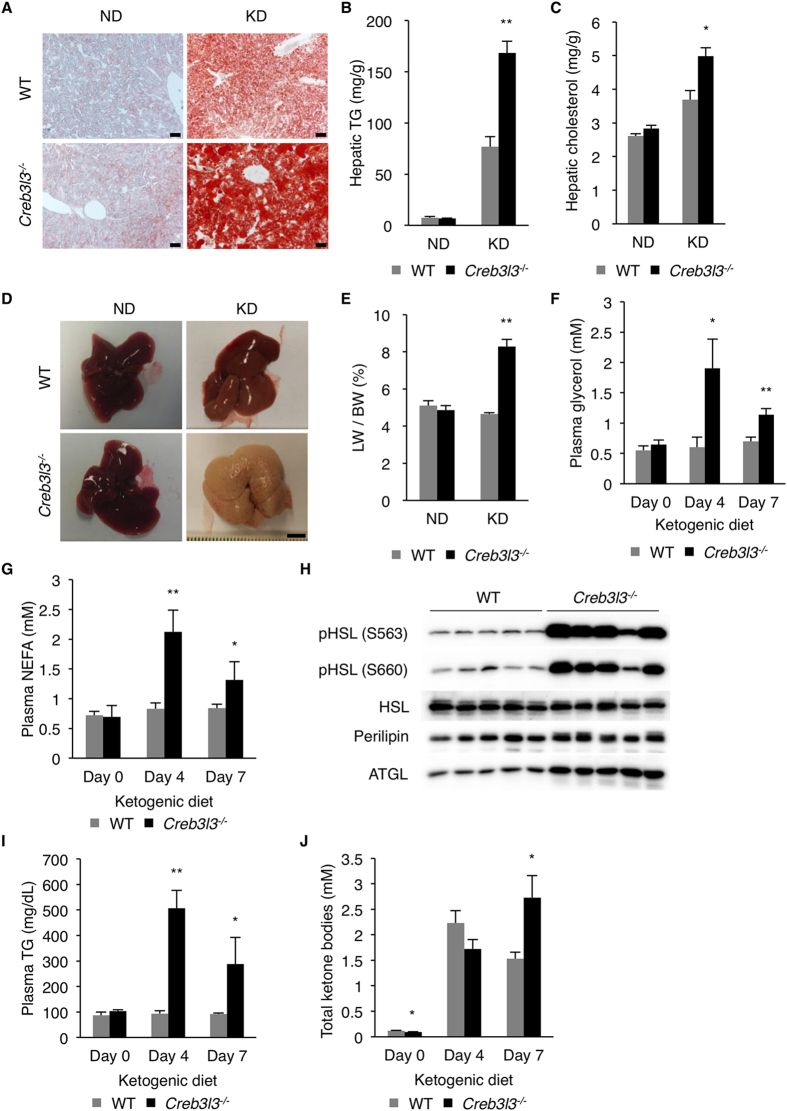
Loss of CREBH exacerbates hepatic steatosis upon ketogenic diet feeding. (**A**) Oil red O staining of liver sections. Mice were sacrificed in the ad libitum fed state. Scale bar = 200 μm. (**B**) Hepatic TG (n = 10 per group), and (**C**) cholesterol levels (n = 3 per group) in mice fed normal chow (ND) or ketogenic diet (KD) for 4 days. (**D**) Liver morphology. (**E**) The ratio of liver weight (LW) to body weight (BW). (n = 10 per group). (**F**) Plasma glycerol and (**G**) NEFA levels (n = 8 per group). (**H**) Western blotting analysis HSL and perilipin phosphorylation, and ATGL expression in WAT. (**I**) Plasma TG, and (**J**) total ketone bodies levels (n = 8 per group). Data are shown as mean ± s.e.m. *P < 0.05, **P < 0.01.

High-fat, low-carbohydrate ketogenic diet (KD) has metabolic effects similar to fasting, shifting the energy source from carbohydrates to fatty acids, which are oxidized in the liver to produce ketone bodies^36^. We tested whether, similarly to fasting, the KD caused severe steatosis in Creb3l3^−/−^ mice. KD caused mild steatosis in WT mice, as determined by oil red O staining of the liver sections and biochemical quantification of hepatic TG and cholesterol contents (Fig. 3A–C). In stark contrast, Creb3l3^−/−^ mice exhibited a dramatic increase in hepatic TG level compared with the WT mice (Fig. 3B). Hepatic cholesterol levels were modestly increased in Creb3l3^−/−^ mice (Fig. 3C). H&E staining confirmed the presence of ballooned hepatocytes with pale cytoplasm in Creb3l3^−/−^ mice, a distinctive feature of hepatic steatosis (Supplementary Fig. 2A). KD also induced hepatomegaly in Creb3l3^−/−^ mice, resulting in a more than 60% increase in liver to body weight ratio (Fig. 3D,E). Creb3l3^−/−^ mice lost more body weight than the WT on KD (Supplementary Fig. 2B). Body weight loss correlated well with the reduced fat mass (Supplementary Fig. 2C). KD dramatically increased plasma glycerol and NEFA levels in Creb3l3^−/−^, but not in WT mice (Fig. 3F,G). Creb3l3^−/−^ mice also exhibited increased HSL and perilipin phosphorylation, and increased ATGL abundance in WAT compared with the WT controls (Fig. 3H), indicating that CREBH deficiency led to dysregulated adipose tissue lipolysis. Plasma TG and ketone body levels measured in the fed state were also markedly higher in Creb3l3^−/−^ than in WT mice on KD (Fig. 3I,J). Interestingly, the expression of PPARα and its target genes was impaired in Creb3l3^−/−^ mice on KD (Supplementary Fig. 2D,E), which might have contributed to the severe steatosis phenotype of these mice. The underlying mechanism for the reduced expression of PPARα in CREBH deficient liver remains to be determined. Lipogenic transcription factors and enzymes were unchanged in Creb3l3^−/−^ mice as compared with WT mice (Supplementary Fig. 2F).

**Figure 4:**
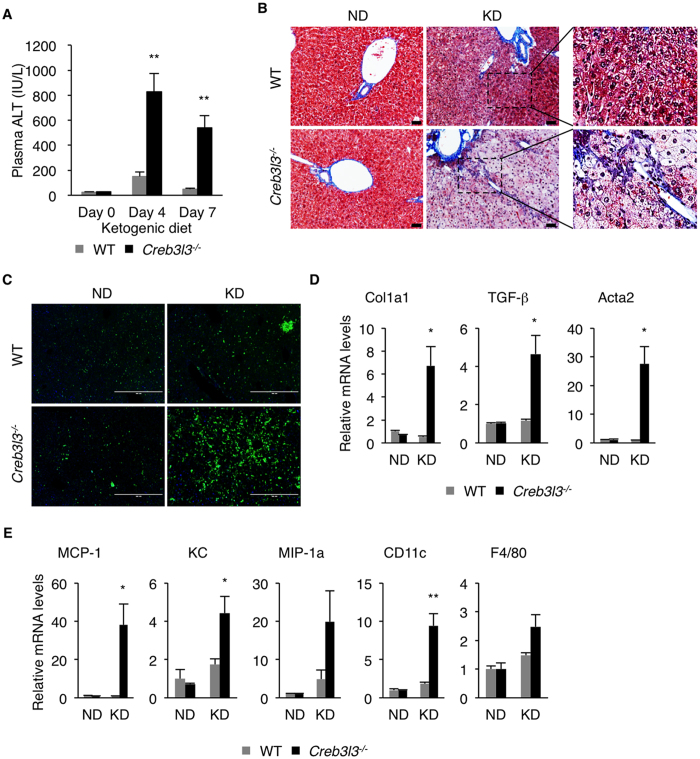
Hepatic inflammation and liver damage in CREBH deficient mice fed ketogenic diet. (**A**) Plasma alanine aminotransferase (ALT) levels un mice fed KD for 0, 4 and 7 days (n = 8 per group). (**B**) Mice were fed ND or KD for 4 days. Liver sections were stained with Masson’s trichrome. Scale bar = 200 μm. (**C**) CD45 immunostaining of liver sections. Scale bar = 400 μm. (**D**) Hepatic mRNA levels of genes related to fibrosis and (**E**) inflammation determined by qRT-PCR (n = 4 per group). Data are shown as mean ± s.e.m. *P < 0.05, **P < 0.01.

Hepatic steatosis often progresses to NASH with associated liver damage, fibrosis, and inflammation. We next investigated whether the severe hepatic steatosis in Creb3l3^−/−^ mice on KD progresses to NASH. KD caused a marked increase in plasma ALT levels in Creb3l3^−/−^ mice, indicating the presence of liver damage (Fig. 4A). Trichrome staining of the liver sections revealed a pronounced deposition of collagen in the liver of Creb3l3^−/−^ mice (Fig. 4B). Infiltration of inflammatory cells was also observed in the liver of Creb3l3^−/−^ mice fed KD (Fig. 4C). Consistent with these histological observations, liver fibrosis-related genes such as Col1a1, TGF-beta1 and Acta2, and inflammatory genes such as MCP-1, KC, MIP-1α, CD11c and F4/80, were highly induced Creb3l3^−/−^ mice (Fig. 4D,E). These data suggest that CREBH deficient mice are highly susceptible to NASH on KD.

### CREBH-induced FGF21 suppresses lipolysis in adipose tissue

**Figure 5:**
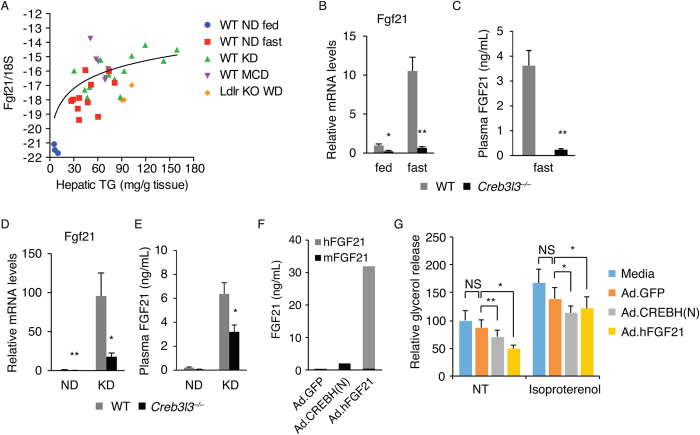
CREBH-induced FGF21 suppresses adipose tissue lipolysis. (**A**) Correlation between hepatic TG and FGF21 mRNA levels on Log_2_ scale in mice. Each symbol represents an individual mouse as indicated. R^2^ = 0.1995, *P* (two-tailed) = 0.0056. (**B**) Hepatic FGF21 mRNA levels determined by qRT-PCR (n = 4 per group). (**C**) Plasma FGF21 levels measured after a 16 h fast (n = 5 per group). (**D**) Hepatic FGF21 mRNA levels (n = 4 per group). (**E**) Plasma FGF21 protein levels (n = 7 per group). (**F**) Total FGF21 concentration in the conditioned media of the primary hepatocytes infected by Ad.GFP, Ad.CREBH(**N**) or Ad.hFGF21. Scale bar = 200 μm. (**G**) *Ex vivo* lipolysis assay. Relative amount of glycerol released from WAT slice cultured in the presence of conditioned media for 3 hours. Isoproterenol (100 nM) was added to culture media to measure stimulated lipolysis. The results are the average of 5–6 independent experiments. A paired t-test was performed for statistical analysis. Data are shown as mean ± s.e.m. *P < 0.05, **P < 0.01.

We next asked how liver-expressed CREBH regulates adipose tissue lipolysis. We reasoned that CREBH might induce a secretory protein(s) that acts on adipocytes to suppress lipolysis. An obvious candidate for this was FGF21, which has been identified as a direct CREBH-target and possesses an anti-lipolytic function^31^,^32^,^37^. In addition, we found that hepatic FGF21 expression was highly increased in various animal models of steatosis, consistent with previous reports^38^,^39^,^40^. Remarkably, there was a strong correlation between hepatic FGF21 mRNA and hepatic TG levels, suggesting that hepatic TG content determines FGF21 transcription (Fig. 5A). Given that CREBH is activated in fatty livers^23^, we hypothesized that fatty livers produce FGF21 via CREBH to suppress lipolysis of adipose tissue, constituting a negative regulatory feedback loop. To test the hypothesis, we first measured hepatic FGF21 mRNA and plasma FGF21 protein levels in WT and Creb3l3^−/−^ mice. Consistent with previous reports^17^,^18^, hepatic FGF21 expression was strongly induced by fasting in WT, but not in Creb3l3^−/−^ mice (Fig. 5B). Plasma FGF21 protein level was also marked lower in Creb3l3^−/−^ than in WT mice (Fig. 5C). KD-induced FGF21 expression was also markedly diminished in Creb3l3^−/−^ mice, indicating a major role of CREBH in hepatic FGF21 expression (Fig. 5D,E). Deletion of both CREBH and PPARα in mice almost completely abolished FGF21 expression induced by fasting (Supplementary Fig. 3A,B), and KD feeding (Supplementary Fig. 3C,D), suggesting that these two transcription factors cooperatively regulate FGF21 transcription in the liver.

We next tested whether FGF21 suppressed lipolysis in an *ex vivo* lipolysis assay. We produced FGF21 in primary mouse hepatocytes by infecting cells with recombinant adenoviruses expressing human FGF21 or CREBH(N), which would induce endogenous FGF21 (Fig. 5F). The conditioned media containing FGF21 were tested for the effects on the rate of glycerol release from adipose tissue slices freshly isolated from overnight fasted mice. Conditioned media from CREBH(N) adenovirus-infected cells containing a moderate level of FGF21 modestly suppressed the glycerol release (Fig. 5G). A high dose of FGF21 in the conditioned media from FGF21 adenovirus-infected cells further suppressed glycerol release, suggesting that FGF21 suppresses adipose tissue lipolysis (Fig. 5G). Glycerol release from isoproterenol-stimulated cells was also reduced by FGF21-containing culture supernatant, suggesting that FGF21 suppressed β-adrenergic stimulated lipolysis (Fig. 5G).

**Figure 6:**
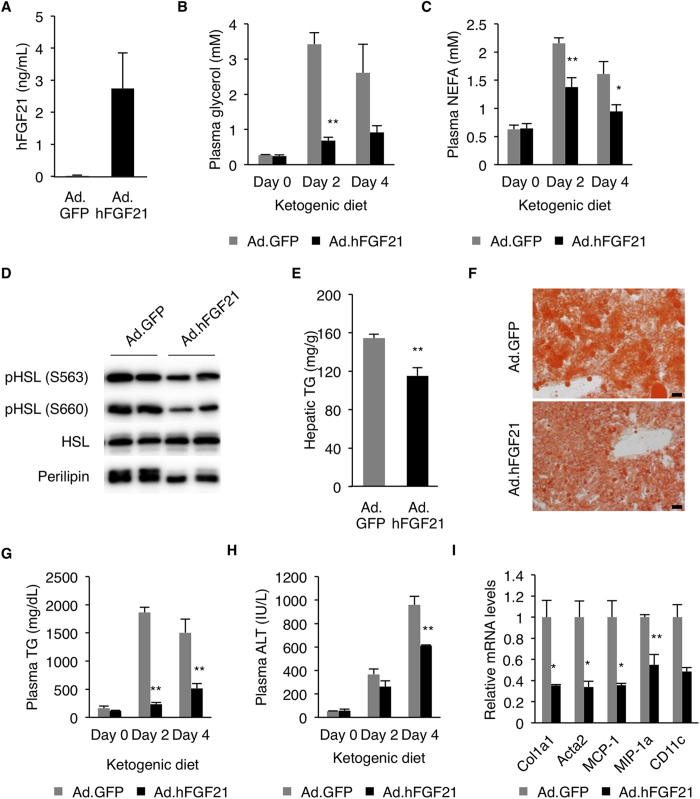
FGF21 improves steatohepatitis of CREBH deficient mice induced by KD. (**A**) Creb3l3^−/−^ mice were infected by Ad.GFP or Ad.hFGF21. Plasma FGF21 levels were measured 2 days after the virus infection (n = 4 per group). (**B**) Plasma glycerol and (**C**) NEFA levels in the adenovirus infected mice measured at days 0, 2 and 4 on KD (n = 4 per group). (**D**) Adipose tissues were isolated from adenovirus-infected Creb3l3^−/−^ mice fed KD for 2 days, and analyzed for HSL and perilipin phosphorylation by western blotting. (**E**) Hepatic TG levels. (**F**) Oil red O staining of liver sections. (**G**) Plasma TG and (**H**) ALT levels (n = 4 per group). (**I**) Hepatic mRNA levels determined by qRT-PCR (n = 4 per group). Data are shown as mean ± s.e.m. *P < 0.05 and **P < 0.01.

We next tested whether FGF21 overexpression ameliorates hepatic steatosis of Creb3l3^−/−^ mice. We injected recombinant adenovirus expressing human FGF21 (Ad.hFGF21) into Creb3l3^−/−^ mice (Fig. 6A), which were then placed on KD 2 days after the viral transduction. FGF21 overexpression markedly decreased plasma glycerol and NEFA levels in Creb3l3^−/−^ mice fed KD (Fig. 6B,C), indicating that FGF21 inhibited lipolysis. HSL and perilipin phosphorylation was also reduced by FGF21 adenovirus in the WAT of Creb3l3^−/−^ mice (Fig. 6D). Hepatic steatosis was improved by FGF21 overexpression in Creb3l3^−/−^ mice, as determined by decreased hepatic TG content and liver size (Fig. 6E,F and Supplementary Fig. 4A). FGF21 also improved the hypertriglyceridemia of Creb3l3^−/−^ mice on KD (Fig. 6G). Interestingly, FGF21 overexpression partially restored the expression of PPARα and its target genes, Acox1, Hmgcs2 and Cpt1a in Creb3l3^−/−^ mice (Supplementary Fig. 4B,**C**), which might have helped to improve the hepatic steatosis. Finally, we analyzed whether FGF21 overexpression ameliorated hepatic fibrosis and inflammation of Creb3l3^−/−^ mice fed KD. Plasma ALT levels were significantly reduced in Ad.hFGF21-injected mice compared with Ad.GFP group (Fig. 6H). Additionally, expression of the inflammatory markers such as Col1al, Acta2, MCP-1, MIP-1α and CD11c, were also reduced by FGF21 overexpression (Fig. 6I). These data suggest that FGF21 is a critical CREBH target that protects against heaptic steatosis and steatohepatitis.

## Discussion

**Figure 7:**
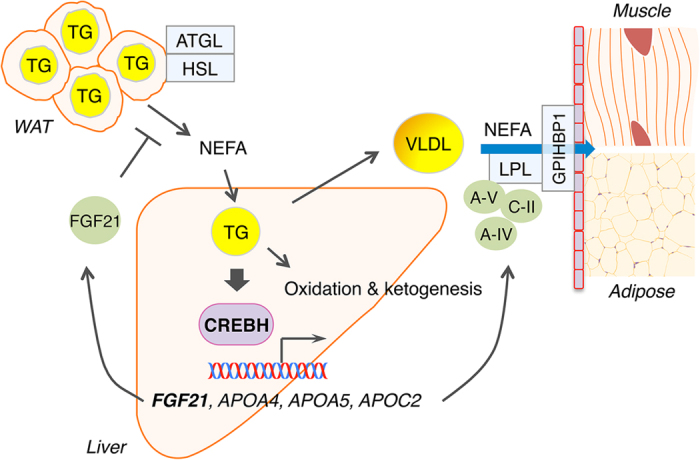
Summary for the role of CREBH in regulating hepatic TG metabolism. CREBH is activated in fatty livers and promotes FGF21 production. FGF21 suppresses lipolysis, alleviating mobilization of TG from adipose tissue to the liver. CREBH also induces apoA-IV, apoA-V and apoC-II that stimulate LPL-mediated TG clearance. Loss of CREBH causes hepatic steatosis and hypertriglyceridemia.

In this study, we demonstrate that CREBH is activated by TG accumulation in the liver and induces FGF21, which suppresses lipolysis in adipose tissue, thereby limiting NEFA flow to the liver (Fig. 7). Impairment of the CREBH-FGF21 axis causes severe steatosis owing to the uncontrolled NEFA flow to the liver in fasted or KD-fed mice. We propose that CREBH acts as a TG rheostat in the liver regulating the flow of fatty acids to the liver. We also show that CREBH deficient mice are susceptible to NASH, identifying CREBH as a potential target for NASH prevention and treatment.

It has been previously reported that CREBH deficient mice are susceptible to hepatic steatosis, but the underlying mechanism remained unclear^17^,^24^. Zhang and colleagues demonstrated that Creb3l3^−/−^ mice fed atherogenic Paigen diet high in saturated fat, cholesterol, and cholate developed severe hepatic steatosis, associated with decreased expression of genes involved in de novo lipogenesis, cholesterol synthesis, fatty acid oxidation, and lipoprotein metabolism^24^. However, it was unclear whether the alteration of hepatic gene expression profile by the loss of CREBH had a causal role in steatosis, or merely represented a consequence of severe steatosis. For example, it is unlikely that decreased expression of lipogenic transcription factors and enzymes contributed to severe steatosis in Creb3l3^−/−^ mice. In the current study, we systematically explored the mechanisms by which CREBH regulates hepatic TG content, and demonstrated that the increased NEFA delivery from adipose tissue to the liver is primarily responsible for the worsened hepatic steatosis in Creb3l3^−/−^ mice. Intriguingly, Zhang *et al*.^24^ showed that fasting plasma ketone levels were lower in Creb3l3^−/−^ than in WT mice, arguing that fatty acid oxidation was impaired in Creb3l3^−/−^ mice. This is in stark contrast to our findings: increased plasma ketone levels in fasted or KD-fed Creb3l3^−/−^ mice compared with WT controls, and comparable fatty acid oxidation activity in liver lysates between the two groups. Currently, we have no explanation for this discrepancy, as both studies used the same strain of Creb3l3^−/−^ mice^41^. Notably, a recent independent study showed that Creb3l3^−/−^ mice had higher ketone levels during starvation compared with the WT controls^42^, consistent with our observations. We speculate that the increased flow of fatty acids from WAT to the liver provides more substrate for ketogenesis, increasing plasma ketone levels in Creb3l3^−/−^ mice.

FGF21 confers multiple metabolic benefits, which include improving hyperglycemia, hyperlipidemia, hepatic steatosis, and obesity^43^. Growing evidence suggests that WAT is a critical target organ responsible for these diverse metabolic effects of FGF21^44^,^45^,^46^. In the current study, we demonstrated that loss of CREBH decreased FGF21 production from the liver, increased adipose tissue lipolysis and worsened hepatic steatosis, which was partially reversed by adenoviral overexpression of FGF21. We also showed that *in vitro* FGF21 suppressed lipolysis in mouse adipose tissue explants freshly prepared from overnight fasted mice, suggesting that CREBH-induced FGF21 suppressed adipose tissue lipolysis to ameliorate hepatic steatosis. It should be noted, however, that the effects of FGF21 on adipose tissue lipolysis is rather complex. An early study showed that FGF21 increased basal, but not isoproterenol-stimulated lipolysis in murine 3T3-L1 adipocytes^47^, whereas a subsequent study found no effect of FGF21 on basal lipolysis under similar experimental conditions^37^. In contrast, recent studies showed that FGF21 suppressed hormone-stimulated lipolysis in both 3T3-L1 and human adipocytes^37^,^48^. FGF21 knockout mice exhibited elevated plasma NEFA levels in fated state^31^, suggesting increased adipose tissue lipolysis in the absence of FGF21, which is consistent with our findings in Creb3l3^−/−^ mice. In contrast, FGF21 injection lowered plasma NEFA in mice, supporting the role of FGF21 in the inhibition of adipose tissue lipolysis^48^,^49^. Interestingly, a recent paper proposed that FGF21 stimulates the flow of fatty acids into adipose tissues, resulting in the reduction of plasma NEFA levels^49^.

FGF21 exerts its biological activity through interaction with a cell surface receptor complex consisting of FGF1R1c and β-Klotho^45^,^50^,^51^,^52^,^53^. FGF receptor activation triggers various intracellular signaling pathways, but the specific pathways activated by FGF21 remains poorly understood^54^,^55^. Creb3l3^−/−^ mice exhibited higher HSL and perilipin phosphorylation, suggesting that FGF21 signaling intersects with PKA pathway. Further study will address intracellular signaling pathways triggered by FGF21 in adipocytes.

Several transcription factors have been implicated in hepatic FGF21 expression^56^, among which PPARα and CREBH appear to play the major role. Loss of PPARα or CREBH almost completely abolished fasting-induced hepatic FGF21 expression in mice^17^,^18^,^38^,^47^. Similarly, the loss of either PPARα or CREBH markedly reduced the induction of hepatic FGF21 by KD, although to lesser degrees than by fasting. Concomitant deletion of PPARα and CREBH further reduced hepatic FGF21 expression in KD-fed mice, indicating that PPARα and CREBH cooperatively regulate FGF21 expression. Consistent with this, a recent study has shown that PPARα and CREBH form a complex that directly binds to FGF21 promoter^18^. The interplay between CREBH and PPARα in the regulation of FGF21 remains to be further elucidated.

The liver is the major source of circulating FGF21 in mice^25^. Early studies in mice revealed that FGF21 is induced by nutrient deprivation^38^,^47^,^57^, however, the effect of prolonged fasting on serum FGF21 protein level in humans is relatively small^58^,^59^. On the other hand, several studies including ours have demonstrated that hepatic FGF21 mRNA and serum FGF21 protein levels are increased in mice and humans with obesity and NAFLD^38^,^39^,^40^, suggesting that TG accumulation may trigger FGF21 expression in the liver. TG accumulation activates CREBH in the liver^23^, which may in turn stimulate FGF21 transcription. In line with this, a recent paper demonstrated that adipose tissue specific CGI-58 knockout mice are protected from fasting-induced hepatic steatosis due to decreased NEFA delivery to the liver, which led to impaired CREBH activation and FGF21 expression^60^. Given that prolonged fasting also induces hepatic steatosis, it is tempting to speculate that the TG accumulation underlies FGF21 expression in fasted mice.

While high concentration of fatty acid ligands in steatotic livers is expected to activate PPARα, it remains unclear how TG accumulation activates CREBH. CREBH mRNA and the nuclear CREBH(N) protein levels are induced in steatotic livers^23^. PPARα appears to play a role in the transcriptional activation CREBH in fatty livers^21^. Since CREBH has to undergo proteolytic cleavage by Golgi proteases for its activation, ER to Golgi transport of CREBH is expected to be a critical regulatory checkpoint. We speculate that the Golgi transport of CREBH might be coupled with TG accumulation or VLDL-apolipoprotein B production in the cell. Given that ER transmembrane transcription factors are associated with specific interacting proteins that regulate the Golgi transport^61^,^62^, it is tempting to speculate that CREBH might also be associated with a yet-to-be identified interaction partner that senses cellular TG level.

The two-hit hypothesis is widely accepted as a mechanism for the progression of simple steatosis to NASH, which involves oxidative stress and pro-inflammatory response in the liver as second hits^63^. KD caused severe hepatocyte death, fibrosis and inflammation in CREBH deficient mice. Interestingly, overexpression of FGF21 ameliorated hepatic steatosis and NASH symptoms of KD-fed Creb3l3^−/−^ mice, suggesting that FGF21 is one of the major CREBH targets that help alleviate steatosis and NASH. Another notable CREBH target that might be involved in lipotoxicity is Fsp27β, which promotes the storage of TG in lipid droplets in hepatocytes, and therefore is expected to reduce lipotoxicity^64^. We speculate that the impaired expression of Fsp27β in CREBH deficient mice might dsiturb TG storage in lipid droplets, exacerbating lipotoxicity and liver danage. Further studies should reveal the contributions of additional CREBH target genes to NAFLD and NASH.

## Materials and Methods

### Animal experiments

Creb3l3^−/−^ mice were backcrossed onto a C57BL/6 background at least 10 times^17^. PPARα knockout mice (B6;129S4-*Ppara*^*tm1Gonz*^/J) were obtained from Jackson laboratory^65^. Mice were housed in a specific pathogen free facility at the Weill Cornell Medical College on a 12h light/dark cycles and fed ad libitum standard chow diet (PicoLab Rodent diet 20, #5058, Lab diet) or ketogenic diet (# F3666, bio-serv). All animal experiments were approved by the Institutional Animal Care and Use Committee at Weill Cornell Medical College (Protocol #2012–0048), and performed in accordance with the approved guidelines.

### Biochemical assays

Plasma TG, glycerol, cholesterol, NEFA, ketone bodies, ALT, insulin, and FGF21 concentrations were determined using assay kits (Serum Triglyceride Determination Kit, Sigma; Amplex Red Cholesterol Assay Kit, Life technologies; NEFA-HR (2), Wako Chemicals; Autokit Total Ketone Bodies, Wako Chemicals; ALT Kit, Bio-Quant; Ultra Sensitive Mouse Insulin ELISA Kit, Crystal Chem; Mouse/Rat FGF-21 Quantikine ELISA Kit, R&D Systems; Human FGF-21 Quantikine ELISA Kit, R&D Systems). Blood glucose levels were measured using Ascensia Breeze 2 Blood Glucose Monitoring System (Bayer). Lipids were extracted from liver tissues with chloroform/methanol mixture (2:1 v/v), as described previously^66^.

### Adenoviruses

Recombinant adenoviruses that expressed human CREBH(N) were generated using pAdTRACK-cmv shuttle vector, as described previously^66^. Human FGF21 adenovirus was purchased from Vector Biolabs. Recombinant adenoviruses were amplified in HEK293 cells and purified using a commercial kit (Virapur). Mice were injected intravenously via tail vein at a doe of 3 × 10^9^ particles of the adenoviruses per g body weight in 0.15 ml of saline.

### Primary hepatocytes isolation and *in vitro* ketogenesis assay

Primary hepatocytes were prepared from male mice at 8–10 weeks of age. Mice were anesthetized with ketamine and xylazine. Livers were perfused with prewarmed liver perfusion medium (17701-038, Life Technologies) for 5 min followed by liver digest medium (17703-034, Life Technologies) for 10 min at 5 ml/min. Isolated hepatocytes (5 × 10^5^ cells/well) were transferred to Primaria 6-well plates (353846, Corning), and cultured in medium 199 (M4530, Sigma) supplemented with 10% fetal bovine serum under an atmosphere of CO_2_ at 37 °C. For *in vitro* ketogenesis, sodium octanoate (2 mM, Sigma, C-5038) was added to culture media. The concentration of total ketone bodies in the culture media was determined 2, 24 and 48 h after the addition of sodium octanoate.

### Glucose tolerance test

Eight- to ten-week-old WT and Creb3l3^−/−^ mice were fasted for 16 h and then injected intraperitoneally with glucose (2 g/kg body weight). Blood glucose concentrations were measured before and 15, 30, 60, 90 and 120 min after glucose administration.

### VLDL secretion assay

VLDL secretion rate *in vivo* was measured as previously described^67^. Briefly, mice were injected with triton WR1339 (500 mg kg^–1^ in saline) via tail vein following a 16 h fasting. Blood samples were drawn at 0, 1, 2, 3, and 4 h after the injection for TG assays.

### Fatty Acid Oxidation Assay

Fatty acid oxidation activity in liver lysates was measured as previously described^68^. Briefly, 50 mg liver tissues were homogenized in 500 μl of 250 mM sucrose. After the lysate was cleared by centrifugation, Triton X-100 was added to the supernatant to a final concentration of 1%. The extract was incubated with a reaction buffer containing 50 mM Tris (pH 8.0), 1 mM DDT, 60 μg/ml BSA, 25 μM palmitoyl-CoA, 200 μM NAD, 1 μM FAD, and 100 μM CoA. NADH concentration in the reaction mixture was spectrophotometrically measured at 340 nm every 30 seconds for 5 min. The rate of NADH generation was calculated, and shown as fatty acid oxidation activity.

### Lipolysis assay

Gonadal fat pads isolated from male mice were cut into small pieces of approximately 50 mg each, and incubated with conditioned media from primary mouse hepatocytes infected with recombinant adenoviruses diluted 1:10 in phenol red-free DMEM. The culture medium was collected 3 hrs later to measure glycerol content using Free Glycerol Reagent (Sigma).

### Histological analysis

Liver pieces were fixed in 10% formalin solution for two hours at room temperature, placed in 20% sucrose overnight at 4 °C, embedded in paraffin, sectioned, and stained with hematoxylin and eosin (H&E). Immunofluorescence staining was performed using CD45 antibody (R&D systems, AF114). Tissue sections were stained with Masson’s trichrome to evaluate fibrosis. For detection of neutral lipid, tissues were embedded in Shandon Cryomatrix (Thermo) and subjected to cryosectioning. Frozen sections were stained with 1.8 g/l Oil Red O solution.

### RNA isolation, and real time PCR

Total RNAs were isolated using TRIZOL Reagents (Life Technologies) according to the manufacturer’s recommendation. Complementary DNAs were generated using the High Capacity cDNA Reverse Transcription kit (Applied Biosystems), and subjected to SYBR-based real-time PCR using the Mx3005P™ system (Agilent Technologies).

### Western blotting

Liver nuclear extracts were prepared as described previously^23^. Western blotting was performed using specific antibodies against ATGL (Cell Signaling, #2138), mouse CREBH^66^, HMGCS (Santa Cruz, sc-33828), HSP90 (Santa Cruz, sc-7947), Lamin B1 (Santa Cruz, sc-56145), HSL (Cell Signaling, #4170), phospho-HSL (Ser563) (Cell Signaling, #4139), phospho-HSL (Ser660) (Cell Signaling, #4126), perilipin (Cell Signaling, #9346), and PPARα (Santa Cruz, sc-9000). Following incubation with secondary antibodies, protein bands were visualized by SuperSignal West Pico chemiluminescence substrate (Thermofisher), imaged using Flurochem E system (Proteinsimple, CA, USA), and quantitated using Alphaview software (Proteinsimple, CA, USA).

## Additional Information

**How to cite this article**: Park, J.-G. *et al*. CREBH-FGF21 axis improves hepatic steatosis by suppressing adipose tissue lipolysis. *Sci. Rep.* **6**, 27938; doi: 10.1038/srep27938 (2016).

## Supplementary Material

Supplementary Information
