# Supplementary material for: CREBH-FGF21 axis improves hepatic steatosis by suppressing adipose tissue lipolysis
Source: Sci Rep. 2016 Jun 15;6:27938. doi: 10.1038/srep27938 (PMC4908383; doi:10.1038/srep27938)
Supplement: Supplementary Information [file srep27938-s1.pdf]

Supplementary information

CREBH-FGF21 axis improves hepatic steatosis by suppressing adipose tissue lipolysis

Jong-Gil Park[1], Xu Xu[1], Sungyun Cho[2], Kyu Yeon Hur[3], Myung-Shik Lee[4], Sander Kersten[5] and Ann-Hwee Lee[1,2,*]

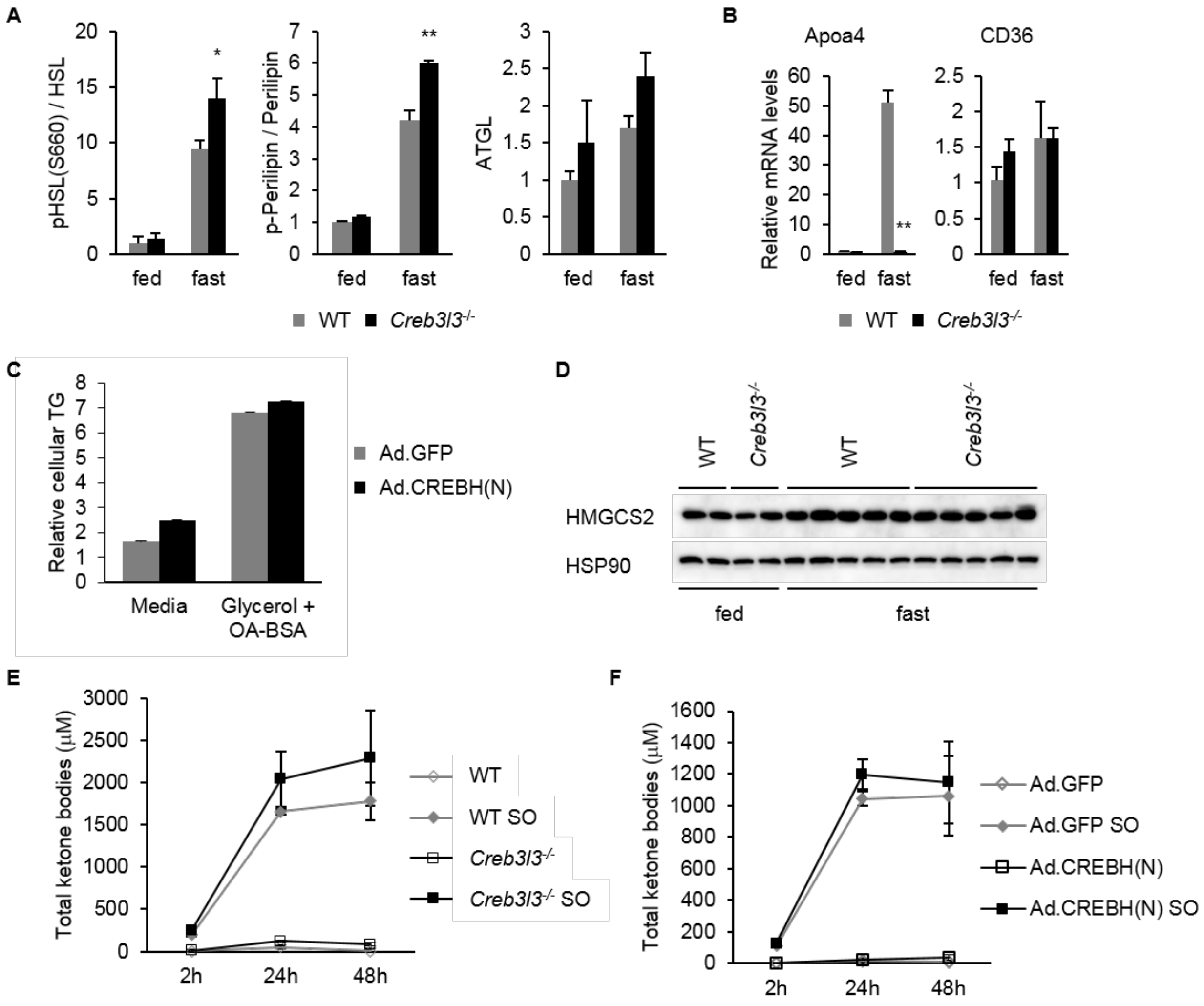

Supplementary Figure 1. Lipogenesis and ketogenesis in CREBH deficient mice. (A) Quantification of western blots shown in Figure 1F. (B) Hepatic mRNA levels determined by qRT-PCR. (n=4 per group). (C) TG synthesis in *Creb3l3* $^{-/-}$ primary hepatocytes infected by Ad.GFP or Ad.CREBH(N) adenoviruses. Cells were cultured in the presence of glycerol (1 mM) and bovine serum albumin-conjugated oleic acid (OA-BSA, 1 mM) for 24 hours. Cellular TG content was measured and normalized to total protein levels. (D) HMGCS2 and HSP90 levels in liver lysates were determined by western blotting. (E) WT or *Creb3l3* $^{-/-}$ primary hepatocytes were cultured in the presence of sodium octanoate (2 mM). Total ketone bodies in the culture media were measured at the indicated time points. (F) Primary mouse hepatocytes were infected with Ad.GFP or Ad.CREBH(N) adenoviruses. Cells were treated with sodium octanoate (2 mM) 24 h after the virus infection, and the culture supernatants were sampled to measure total ketone bodies. Data are shown as mean ± s.e.m. ***P* < 0.01.

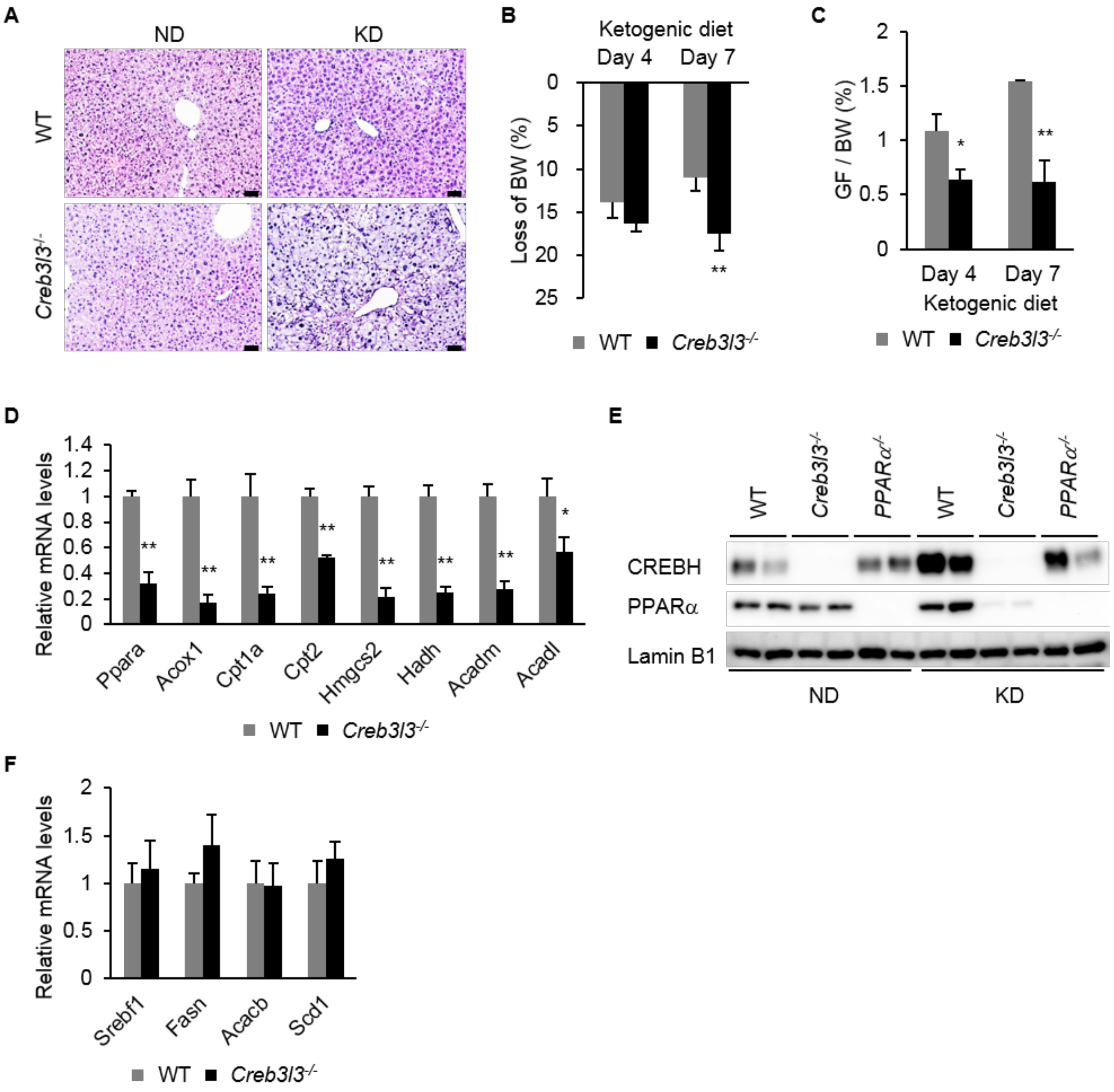

Supplementary Figure 2. Effects of ketogenic diet in CREBH deficient mice. (A) Hematoxylin and eosin staining of liver sections. Scale bar = 200 μm. (B) Loss of body weight (BW) by KD consumption (n=10 per group), and (C) the ratio of gonadal fat (GF) to BW (n=5 per group). (D) Hepatic mRNA levels of PPARα and its target genes determined by qRT-PCR (n=4 per group). (E) Liver nuclear extracts were subjected to western blotting analysis using PPARα, CREBH and Lamin B1 antibodies. A representative image of 3 independent experiments is shown. (F) Hepatic mRNA levels of lipogenic genes determined by qRT-PCR (n=4 per group). Data are shown as mean ± s.e.m. $^*P < 0.05$, $^{**}P < 0.01$.

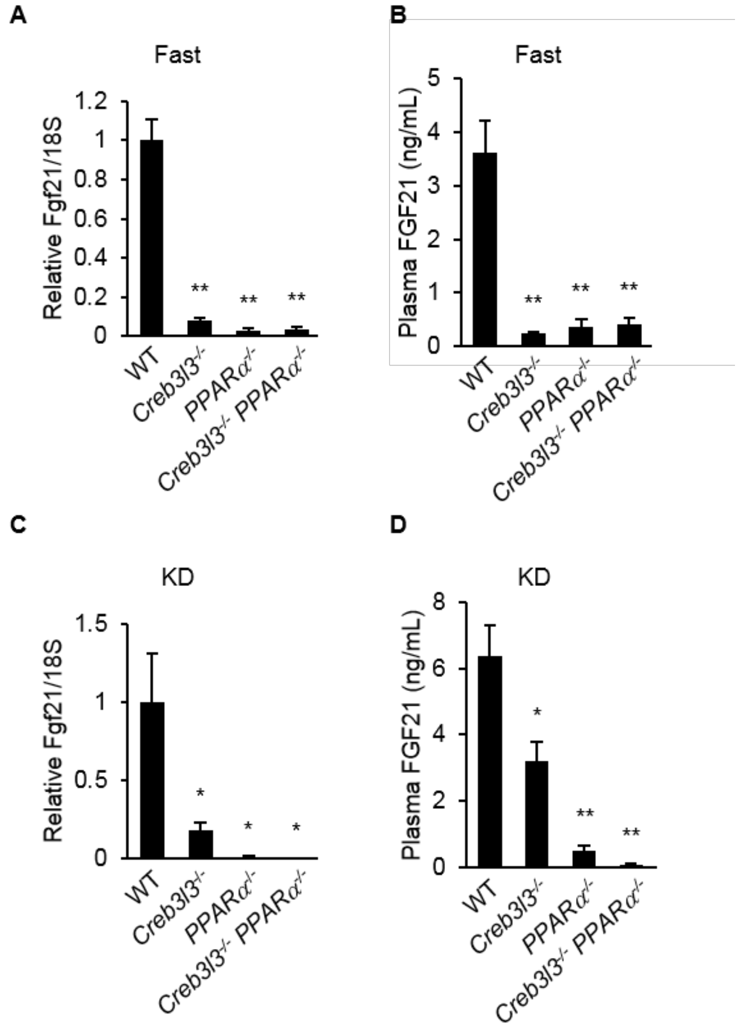

Supplementary Figure 3. Cooperative regulation of FGF21 expression by PPARα and CREBH (A) Fasting-induced hepatic FGF21 mRNA (n=4 per group) and (B) serum FGF21 protein (n=5 per group) in WT, *Creb3l3* $^{-/-}$, *PPARα*$^{-/-}$ and *Creb3l3* $^{-/-}$ *PPARα*$^{-/-}$ double knockout mice. (C) Hepatic FGF21 mRNA (n=4 per group), and (D) plasma FGF21 protein levels (n=7 per group) in mice fed ketogenic diet for 4 days.

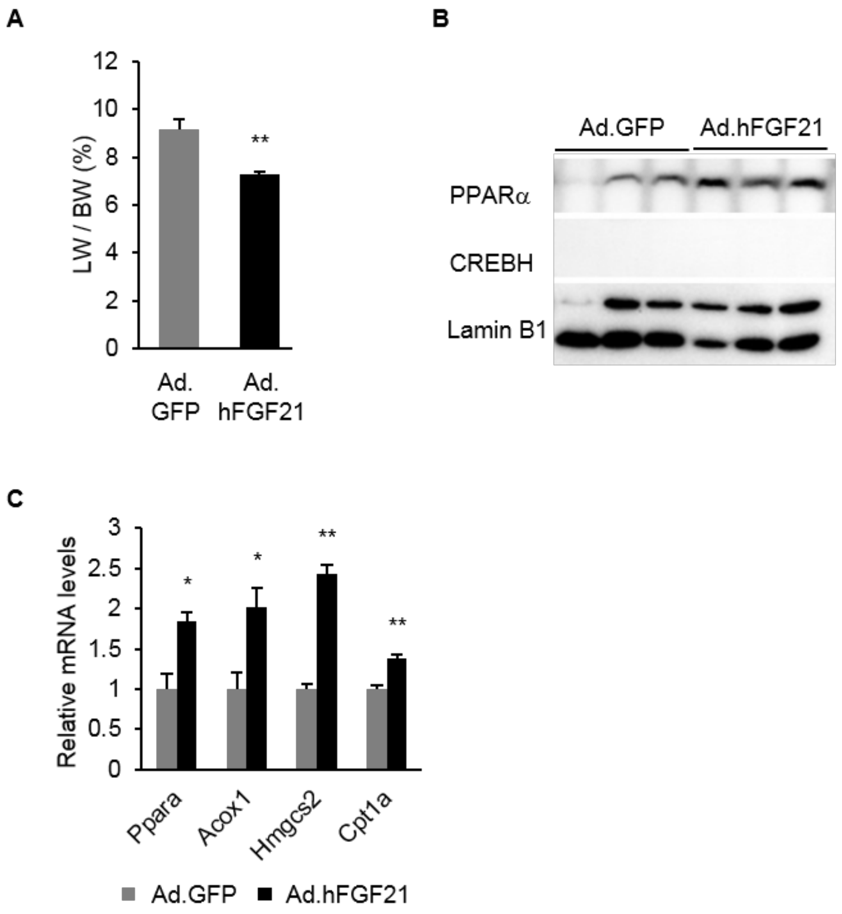

Supplementary Figure 4. FGF21 overexpression induces PPARα and its target genes in CREBH deficient mice. (A) *Creb3l3*$^{-/-}$ mice were infected by Ad.GFP or Ad.hFGF21, and fed ketogenic diet for 4 days. Liver weight (LW) to body weight (BW) ratio was determined (n=4 per group). (B) Liver nuclear extracts of the adenovirus-infected mice were subjected to western blotting using PPARα, CREBH and Lamin B1 antibodies. (C) Hepatic mRNA levels of PPARα and its target genes determined by qRT-PCR. (n=4 per group). Data are shown as mean ± s.e.m. *$P < 0.05$, **$P < 0.01$.
